# Blood flow changes in the forearm arteries after ultrasound-guided costoclavicular brachial plexus blocks: a prospective observational study

**DOI:** 10.1186/s12871-021-01383-w

**Published:** 2021-05-29

**Authors:** Yang Xu, Derong Cui, Junfeng Zhang, Qian Ding, Jing Dong, Yan Wang

**Affiliations:** Department of Anesthesiology, Shanghai Jiaotong University Affiliated Sixth People’s Hospital, 600 Yishan Road, Shanghai, China

**Keywords:** brachial plexus block, costoclavicular block, radial artery, ulnar artery, blood flow

## Abstract

**Background:**

An increase in blood flow in the forearm arteries has been reported after brachial plexus block (BPB). However, few studies have quantitatively analysed the blood flow of the forearm arteries after BPB or have studied only partial haemodynamic parameters. The purpose of the present study was to comprehensively assess blood flow changes in the distal radial artery (RA) and ulnar artery (UA) after BPB performed via a new costoclavicular space (CCS) approach using colour Doppler ultrasound.

**Methods:**

Thirty patients who underwent amputated finger replantation and received ultrasound-guided costoclavicular BPB were included in the study. The haemodynamic parameters of the RA and UA were recorded before the block and 10 min, 20 min, and 30 min after the block using colour Doppler ultrasound to determine the peak systolic velocity (PSV), end-diastolic velocity (EDV), mean velocity (V_mean_), pulsatility index (PI), resistance index (RI) and area. The volumetric flow rate (VFR) was calculated using the formula Q = area×V_mean_. The aforementioned parameters were compared not only before and after the BPB but also between the RA and UA.

**Results:**

Compared with those of the respective baselines, there was a significant increase in the PSV, EDV, V_mean_, area, and VFR and a significant decrease in the PI and RI of the RA and UA 10 min, 20 min, and 30 min post-block. The increase 30 min post-block in EDV (258.68 % in the RA, 279.63 % in the UA) was the most notable, followed by that in the V_mean_ (183.36 % in the RA, 235.24 % in the UA), and the PSV (139.11 % in the RA, 153.15 % in the UA) changed minimally. The V_mean_ and VFR of the RA were significantly greater than those of the UA before the BPB; however, there was no significant difference in the VFR between the RA and UA after the BPB.

**Conclusions:**

A costoclavicular BPB can increase blood flow in the forearm arteries. The RA had a higher volumetric flow rate than the UA before the BPB; however, the potential blood supply capacity of the UA was similar to that of the RA after a BPB.

**Trial registration:**

This study was registered at the Chinese Clinical Trial Registry (http://www.chictr.org.cn/searchproj.aspx, clinical trial number: ChiCTR 1900023796, date of registration: June 12, 2019)

## Background

Studies have reported that a brachial plexus block (BPB) can provide not only local analgesia but also sympathetic blockade, which causes vasodilatation and increases blood flow in the forearm arteries [1–6]. This anti-sympathetic effect is of great importance in preventing vasospasm after vascular reconstruction and replantation.

The traditional lateral sagittal infraclavicular BPB can provide anaesthetic effects for forearm surgery [7]. However, this technique is limited in that the three cords of the brachial plexus are located deep within the pectoral muscles and are scattered around the axillary artery (AA) with a highly variable position. Therefore, a large dose (up to 35–40 mL) of local anaesthetic and multiple injections are required to produce a successful brachial plexus blockade [8, 9]. Recently, a new approach for infraclavicular BPB via the costoclavicular space [10] (CCS) was proposed. At the CCS, the three cords of the brachial plexus are clustered together between the subclavius and serratus anterior muscles and are consistently lateral to the AA. Hence, a single injection with a smaller volume of local anaesthetic (20–25 mL) at the centre of the nerve cluster produces a blockade of the terminal nerves of the brachial plexus.

The increase in blood flow in the forearm arteries after BPB results from BPB playing a sympatholytic role in preventing vasospasm. However, few studies have quantitatively analysed the blood flow of the forearm arteries after BPB or have studied only partial haemodynamic parameters. The current prospective study aimed to comprehensively determine the blood flow parameters of the distal radial artery (RA) and the ulnar artery (UA) after costoclavicular BPB using colour Doppler sonography.

## Methods

### Study design

This prospective observational prospective study was registered in the Chictr.org.cn registry system on 12 June 2019 (ChiCTR1900023796) and was approved by the institutional ethics committee (Approval Number: 2019-060-1). This study was conducted in Shanghai Sixth People’s Hospital from September 2019 to March 2020.

### Inclusion and exclusion criteria

After obtaining written informed consent, 32 patients aged 18 to 60 years with American Society of Anesthesiologists (ASA) physical status I to II who were to undergo emergent finger replantation were included. Patients who had a history of coagulation disorders, peripheral arterial disease, heart disease, diabetes and hypertension were excluded from the study.

### Interventions

**Fig. 1:**
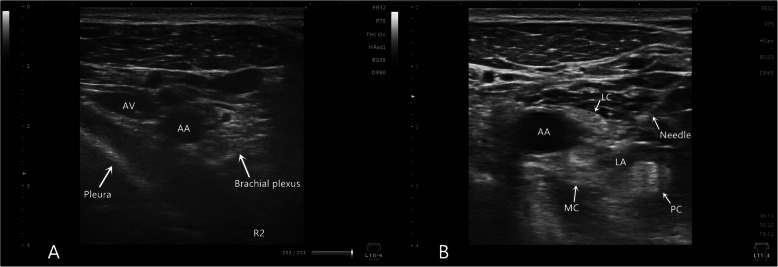
**a** Ultrasound image of the brachial plexus at the CCS. The three cords of the brachial plexus are clustered together lateral to the AA, which shares a consistent relation with the AA. **b** Ultrasound image of the brachial plexus block at the CCS. The needle tip is placed at the centre of the nerve cluster by advancing the needle through the gap between the LC and PC and advancing it towards the MC. Ropivacaine is injected at the centre of the nerve cluster and surrounds the 3 cords as a local anaesthetic AA, axillary artery; AV, axillary vein; LC, lateral cord; MC, medial cord; PC, posterior cord; R2, second rib; LA, local anaesthetic; CCS: costoclavicular space

After standard monitoring including noninvasive blood pressure, electrocardiography and peripheral oxygen saturation (SpO_2_), ultrasound-guided (Sonimage MX1, Konica Minolta, Japan) costoclavicular BPB was performed. The patient was placed in a supine position with the arms abducted at 90 degrees, and a soft padding was placed on the back. Following implementation of strict aseptic precautions, a high-frequency linear array probe was placed over the midpoint of the clavicle in the transverse orientation. Then, the transducer was moved caudally until the axillary artery and vein were visualized as it left the inferior border of the clavicle. All 3 cords of the brachial plexus were lateral to the axillary artery, which was located between the posterior surface of the clavicle and the second rib (Fig. 1 a). An 18-gauge needle was inserted in-plane in a lateral-to-medial direction after 2 ml of 1 % lidocaine was injected subcutaneously. The needle tip was placed at the centre of the nerve cluster by advancing the needle through the gap between the lateral and posterior cord and advancing it towards the medial cord. Then, 25 ml of 0.5 % ropivacaine was injected at the centre of the nerve cluster to surround the 3 cords with local anaesthetic under sonographic imaging (Fig. 1b). A complete block was defined as a loss of sensation to needle pricks in the area of the cutaneous distribution of the radial nerve, median nerve, ulnar nerve and musculocutaneous nerve.

### Outcomes

**Fig. 2:**
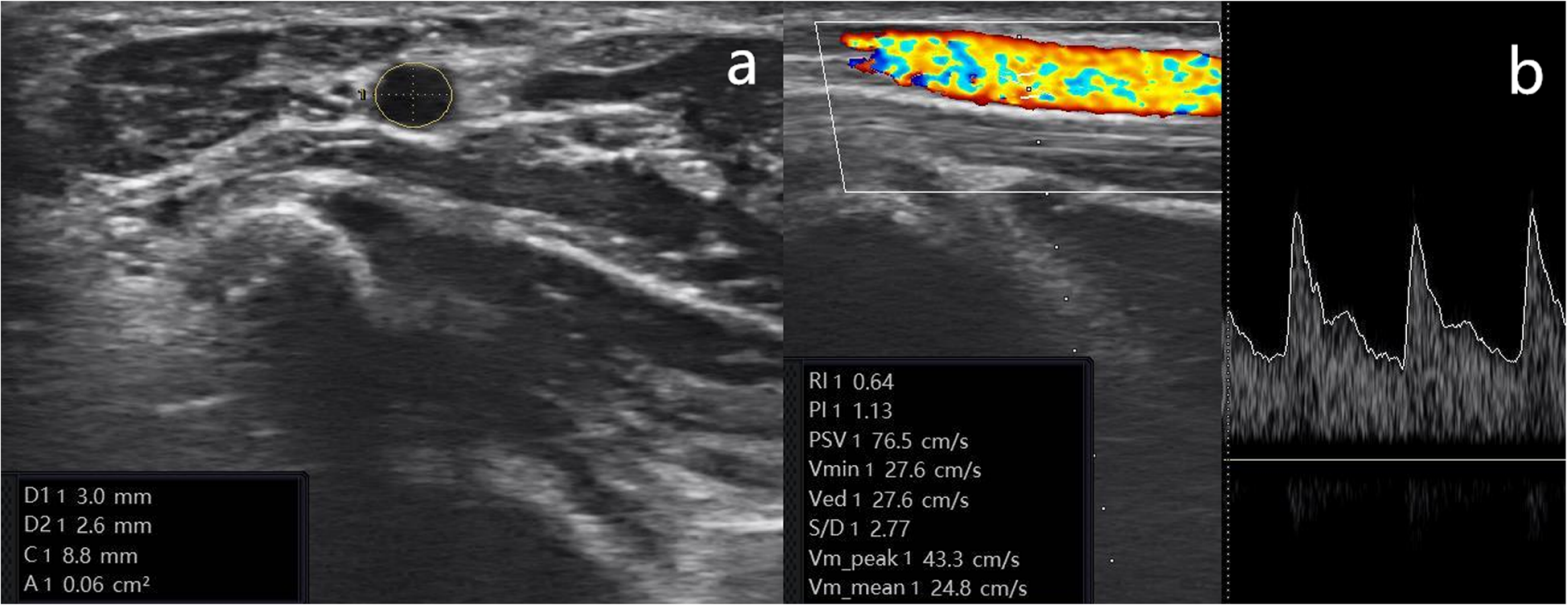
**a** Ultrasound image used to measure the UA cross-sectional area. **b** Longitudinal duplex Doppler spectral waveform for the UA UA, ulnar artery

The peak systolic velocity (PSV), end-diastolic velocity (EDV), mean velocity (V_mean_), pulsatility index (PI), resistance index (RI) and area of the RA and UA were measured at the level of the wrist in the distal forearm by colour Doppler ultrasound before the block and 10 min, 20 min, and 30 min after the block. During the measurements, the patient was placed in the supine position with the arm abducted 90 degrees. All measurements were obtained in the operating room, and the room temperature was controlled at approximately 23 °C. The volumetric flow rate (VFR) was calculated using the formula VFR = area×V_mean_. The examination point was marked one centimetre proximal to the radial-ulnar styloid process to ensure consistency among all measurements taken. Colour duplex Doppler ultrasound (Sonimage MX1, Konica Minolta, Japan) with a broadband 12 − 5 MHz linear array probe was used to image the vessels; the ultrasound scans were performed by two experienced physicians who had more than 10 years of experience with ultrasound imaging. One physician performed the ultrasound examination of the patients, while the other adjusted the equipment parameters and obtained the measurements. To maximize the intensity of the signal, the Doppler angle was maintained at 60°. A short-axis ultrasound image of the artery was obtained to calculate the artery cross-sectional area when the probe was placed at the examination point perpendicular to the artery (Fig. 2 a and b). A long-axis ultrasound image of the artery was obtained by placing the transducer’s midpoint at the examination point, parallel to the artery, and was used to measure the flow velocity (Fig. 2 c and d). The artery cross-sectional area (cm^**2**^) and flow velocity (cm/s) were measured using manufacturer-installed software.

### Sample size

The sample size was calculated based on our pre-trial sample of 10 patients in August 2019, among whom the mean standard deviations of PSV, EDV, MV, RI and PI of the forearm arteries before brachial plexus block were 7.56 cm/s, 1.65 cm/s, 4.56 cm/s, 0.12 and 0.58, respectively, while those after block were 11.68 cm/s, 7.93 cm/s, 7.50 cm/s, 0.11 and 0.44, respectively. A minimum of 32 patients was determined by using a standard sample size calculation formula to achieve a power of 0.8 at α = 0.05, taking into account a 20 % increase for those who might experience an imperfect nerve block effect.

### Statistical analysis

Comparisons of changes in the haemodynamic parameters (PSV, EDV, V_mean_, RI, PI and VFR) of the RA and UA were assessed by the independent-samples t test. A *P* value < 0.05 was considered statistically significant. Normally distributed quantitative variables are expressed as the mean ± standard deviation. SPSS for Windows version 23.0 (SPSS Inc., Chicago, Illinois) was used for statistical analysis and graphing.

## Results

### Demographic data of the patients

A total of 32 patients scheduled to undergo finger replantation signed informed consent forms to participate in the study. After ultrasound-guided costoclavicular BPB, failure of ulnar nerve blockade occurred in 2 cases. The blockades of the radial nerve, ulnar nerve, median nerve and musculocutaneous nerve for the remaining 30 patients were effective. A description of the demographic data is presented in Table 1.

**Table 1:** Patient demographic data

**Characteristics**	**Patients **(*n* = 30)
Mean age (SD) in years	39.3 (18.5)
Sex, n (%)	
Male	22 (73.3)
Female	8 (26.7)
Mean BMI (SD) in kg/m[2]	26.5 (4.3)
ASA classification, n (%)	
I	18 (60.0)
II	12 (40.0)
Replantation Digit, n (%)	
D I	11 (36.7)
D II	10 (33.3)
D I+II	6 (20)
D I+II+III	2 (6.7)
D II+III+IV	1 (3.3)

Data are presented as the mean ± SD. Categorical variables are expressed as patient numbers (%) or numbers

*BMI* body mass index, *ASA* American Society of Anesthesiologists, D I–VI affected fingers

### Comparison of haemodynamic parameters before and after BPB

**Fig. 3:**
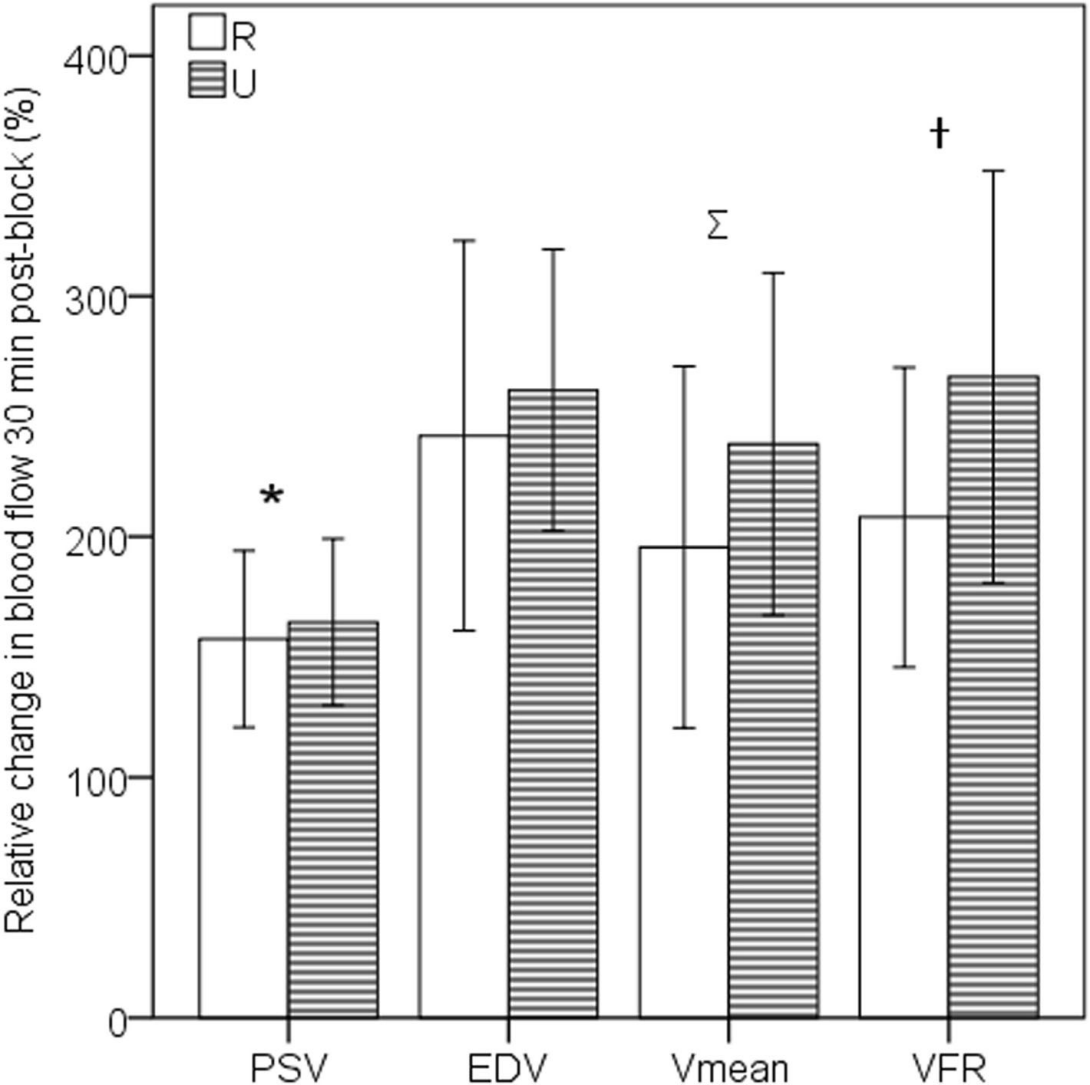
Relative changes in the PSV, V_mean_ and EDV 30 min post-block measured in the RA and UA *P < 0.01 when comparing the relative change in the PSV with that in the EDV. Ψ*P* < 0.05 when comparing the relative change in Vmean of the RA with that of the UA. †*P* < 0.05 when comparing the relative change in the VFR of the RA with that of the UA PSV, peak systolic velocity; EDV, end-diastolic velocity; Vmean, mean velocity; VFR, volumetric flow rate

Changes in the haemodynamic parameters of the RA and UA before and after BPB are summarized in Table 2. The relative changes in blood flow between baseline and 30 min post-block in the RA and UA are shown in Fig. 3. The increase in PSV from baseline to 30 min post-block was 22.50 (7.45) cm/s (*P* < 0.001) for the RA and 23.36 (8.12) cm/s for the UA (*P* < 0.001). The increase in EDV from baseline to 30 min post-block was 14.21 (4.39) cm/s (*P* < 0.001) for the RA and 13.86 (4.14) cm/s for the UA (*P* < 0.001). The increase in V_mean_ from baseline to 30 min post-block was 17.63 (3.91) cm/s (*P* < 0.001) for the RA and 15.13 (3.25) cm/s for the UA (*P* < 0.001). The increase in area from baseline to 30 min post-block was 0.013 (0.0032) cm^2^ (*P* < 0.05) for the RA and 0.016 (0.0045) cm^2^ for the UA (*P* < 0.05). The decrease in the RI from baseline to 30 min post-block was 0.15 (0.040) (*P* < 0.01) for the RA and 0.16 (0.057) for the UA (*P* < 0.01). The decrease in the PI from baseline to 30 min post-block was 1.12 (0.25) (*P* < 0.01) for the RA and 1.21 (0.30) for the UA (*P* < 0.01). The increase in VFR from baseline to 30 min post-block was 1.12 (0.23) ml/s (*P* < 0.001) for the RA and 1.14 (0.34) ml/s for the UA (*P* < 0.001). There was no significant difference among the haemodynamic parameters at 10 min, 20 min, and 30 min after BPB for the RA or the UA. The relative change in the PSV (139.11 % in RA, 153.15 % in UA) was significantly lower than that of the V_mean_ (183.36 % in RA, 235.24 % in UA) (*P* < 0.01), while the relative change in the EDV (258.68 % in RA, 279.63 % in UA) was significantly greater (*P* < 0.05) than that of the V_mean_ for both the RA and UA (Fig. 3).

### Comparison of the haemodynamic parameters between the RA and UA

Of all the parameters, the V_mean_ and VFR of the RA were greater (*P* < 0.05) than those of the UA before the BPB (Table 2). However, no significant difference was found between the haemodynamic parameters of the RA and UA after BPB. The relative change in the V_mean_ and VFR of the UA between baseline and 30 min post-block was significantly greater than that of the V_mean_ and VFR of the RA, respectively (*P* < 0.05; Fig. 3). The difference in the relative change in the PSV and EDV between the RA and UA was not significant.

**Table 2:** Haemodynamic parameters of the RA and UA before and after costoclavicular BPB

					*P Value*				
					Vs. baseline			Vs. UA	
	baseline	10 min	20 min	30 min	10 min	20 min	30 min	0 min	30 min
RA									
PSV (cm/s)	36.68 (7.26)	55.07 (12.28)	57.26 (13.96)	58.97 (13.86)	<0.001	<0.001	<0.001	NS	NS
EDV (cm/s)	9.17 (1.71)	19.69 (7.45)	20.94 (8.63)	21.76 (7.11)	<0.001	<0.001	<0.001	NS	NS
V_mean_(cm/s)	16.38 (4.87)	27.20 (7.74)	28.43 (7.40)	29.58 (7.32)	<0.001	<0.001	<0.001	0.042	NS
Area (cm^2^)	0.056 (0.011)	0.063 (0.012)	0.065 (0.014)	0.068 (0.015)	0.033	0.026	0.012	NS	NS
RI	0.87 (0.11)	0.74 (0.12)	0.73 (0.10)	0.72 (0.11)	0.0058	0.0032	0.0027	NS	NS
PI	3.21 (0.60)	2.35 (0.49)	2.35 (0.43)	2.25 (0.37)	0.0028	0.0012	0.0017	NS	NS
VFR (ml/s)	0.92 (0.33)	1.70 (0.58)	1.87 (0.66)	2.01 (0.72)	<0.001	<0.001	<0.001	0.026	NS
UA									
PSV (cm/s)	35.29 (8.64)	53.76 (11.98)	56.33 (10.79)	58.20 (10.72)	<0.001	<0.001	<0.001		
EDV (cm/s)	8.82 (2.24)	18.69 (7.50)	20.44 (8.69)	22.12 (6.91)	<0.001	<0.001	<0.001		
V_mean_(cm/s)	13.38 (5.06)	26.74 (6.67)	28.41 (7.59)	29.72 (7.97)	<0.001	<0.001	<0.001		
Area (cm^2^)	0.054 (0.011)	0.063 (0.010)	0.065 (0.010)	0.066 (0.014)	0.032	0.016	0.019		
RI	0.87 (0.10)	0.75 (0.10)	0.73 (0.13)	0.72 (0.12)	0.0074	0.0047	0.0021		
PI	3.10 (0.45)	2.22 (0.36)	2.10 (0.38)	2.07 (0.42)	0.0086	0.0046	0.0053		
VFR (ml/s)	0.72 (0.28)	1.68 (0.51)	1.83 (0.52)	1.95 (0.62)	<0.001	<0.001	<0.001		

Data are presented as the mean ± SD

*RA* Radial artery, *UA* Ulnar artery, *PSV* Peak systolic velocity, *EDV* End-diastolic velocity, *V*_mean_ Mean velocity, *PI* Pulsatility index, *RI* resistance index, *VFR* Volumetric flow rate, *NS* not significant

## Discussion

The interscalene, supraclavicular, infraclavicular and axillary approaches are commonly used for BPB and have been reported to achieve sympathetic blockade and vasodilation, which leads to increased blood flow in the forearm arteries [1–6]. In this study, the haemodynamic data of the radial and ulnar arteries of the forearm after costoclavicular BPB were obtained more comprehensively than in other studies due to the measurement of the vascular area and calculation of the VFR. After BPB, blood flow velocity and area increased, while the resistance and pulsatility indexes decreased. The changes in these parameters eventually lead to a significant increase in blood flow. It should be noted that from 10 to 30 min after the BPB, the haemodynamic parameters in the RA and UA did not change significantly over time, indicating that the sympathetic block had a capsulizing effect. Furthermore, the relative changes between baseline and 30 min post-block were compared among the PSV, EDV and V_mean_ to investigate the influence of BPB on blood flow velocity in the RA and UA. The increase in EDV was greater than that of the PSV, which could be explained by the fact that the EDV and V_mean_ reflect vascular compliance and distal vascular resistance, which can partly reflect the degree of sympathetic block. However, the PSV is mainly affected by cardiac function and vascular anatomy, so BPB has a greater influence on EDV than on PSV.

Although it was found that the sympathetic nerves could be blocked after BPB, there is a lack of systematic data on the sympathetic innervation of the upper limbs, and those that have been published are discrepant. Unlike the somatic nervous system, which consciously controls skeletal muscles, the autonomic nervous system controls cardiovascular and smooth muscles using afferent information from visceral organs in an unconscious manner. The autonomic nervous system is divided into the sympathetic and parasympathetic systems, which tend to have opposing effects on the innervated organs and systems. The tissue of the limbs is regulated by sympathetic nerves and lacks parasympathetic innervation [11]. The sympathetic nerves innervating the upper limb originate from two postganglionic neurons: one originates from thoracic ganglia 2–6, located in the sympathetic trunk, and the other is from the stellate ganglion, which forms via fusion of the inferior cervical ganglion and the first thoracic ganglion [12]. The fibres from these two postganglionic neurons join the spinal nerves, which form the brachial plexus and travel through the tissues of the upper limb as sympathetic nerves [13]. The sympathetic nerves in the upper limbs separate from the mixed nerves to create a zonal segmental structure of innervation for the vessels and skin. Unlike somatic fibres, the fibres involved in sympathetic innervation do not show a distinct segmental and neurospecific distribution. Fluorescence microscopy of catecholamine-containing tissues has shown that sympathetic fibres are mainly present in the peripheral areas of the forearm and digital nerve and that those in the median nerve are more abundant than those in the ulnar nerve [14]. Skin temperature measured by infrared thermography after nerve block substantially increases in areas of the hand following specific blockade of the ulnar and median nerves. However, the specific blockade of the musculocutaneous or radial nerve does not increase the temperature in any area [15]. Combined with the above evidence, we believe that the sympathetic fibres of the forearm do not pass through the radial nerve and the musculocutaneous nerve but are contained within the median nerve and the ulnar nerve.

In this study, we compared the haemodynamic parameters of the RA and UA before and after BPB. There was no significant difference between the area of the RA and that of the UA, either before or after BPB. This is consistent with the results of an ultrasonography study conducted by Sunil on the size of the distal RA and UA in 204 patients in southern Rajasthan [16]. Recently, debate has arisen regarding whether the dominant artery for the hand blood supply is the RA or the UA. The UA is considered to be the dominant artery for the blood supply of the forearm and hand according to classic anatomical studies; [17] therefore, the RA is typically used for invasive procedures such as arterial catheterization, coronary artery bypass surgery and arteriovenous (AV) fistula formation for haemodialysis. However, Haerle’s study revealed that the ulnar artery was dominant only to the elbow, while the radial artery was the dominant artery in the distal forearm [18]. In this study, the VFR of the RA was significantly greater than that of the UA under normal circumstances (i.e., preblock), indicating that the RA is the dominant artery for the hand blood supply, which is consistent with the conclusion of Haerle’s study. However, the difference in the VFR between the two arteries disappeared after BPB due to the significant increase in the V_mean_ of the UA, indicating that the UA’s potential blood supply capacity is similar to that of the RA because of its compensatory capacity.

In this study, failure of ulnar nerve block occurred in 2 patients; we consider this to be due to the localization of the medial cord, which is deeper and closer to the axillary artery at the location of the injection used in this approach. We believe that liquid separation of the local anaesthetic applied between the medial cord and the axillary artery may make the blocking more effective. Nevertheless, the advantage of the CCS approach over the conventional approach is still important because the three cords of the brachial plexus are lateral to the axillary artery, which is located superficially. Therefore, because there is no need to frequently change the direction of the needle with the CCS approach, it is easier for doctors who lack experience with the infraclavicular BPB approach to perform it, minimizing tissue damage.

Continuous brachial plexus block has been found to be effective in postoperative pain management and achievement of a sympathetic blockade, which can improve circulation in the replantation finger, prevent blood clots and reduce the incidence of vasospasm after replantation [19–21]. However, some problems remain with this technique, such as an imperfect blockade and the need for catheter fixation [21]. Considering that the three cords of the brachial plexus are scattered around the axillary artery at the location used for the lateral sagittal infraclavicular approach and that the supraclavicular approach can make catheterization difficult due to the bulge of the clavicle, the CCS approach is theoretically the preferred approach for a continuous brachial plexus block. This approach only requires a single point puncture to block the four branches of the brachial plexus, providing effective postoperative analgesia for surgery in any part of the forearm, while simultaneously, the catheter can be fixed to the flat area of the infraclavicular skin to prevent detachment. Therefore, in future research, we will study blood flow changes in the forearm arteries after continuous costoclavicular brachial plexus blocks with lower concentrations of ropivacaine (0.2 %).

## Conclusions

The new costoclavicular BPB approach can reduce peripheral vascular resistance and increase the blood flow in the forearm arteries. The increase in the EDV was the most notable, followed by that in V_mean_, and the PSV changed minimally. The RA had a higher volumetric flow rate than the UA before BPB; however, the potential blood supply capacity of the UA was similar to that of the RA after BPB.

## Data Availability

All the data used and analysed are available from the corresponding authors upon reasonable request.

## Abbreviations

BPB: Brachial plexus block
AA: Axillary artery
CCS: Costoclavicular space
RA: Radial artery
UA: Ulnar artery
PSV: Peak systolic velocity
EDV: End-diastolic velocity
V_mean_: Mean velocity
PI: Pulsatility index
RI: Resistance index
VFR: Volumetric flow rate
ASA: American society of anesthesiologists
SpO_2_: Peripheral oxygen saturation
